# Glucagon-like peptide−1 receptor agonist use and risk of postmastectomy lymphedema in breast cancer survivors: a multicenter real-world cohort study

**DOI:** 10.1007/s00520-026-11405-3

**Published:** 2026-10-27

**Authors:** Dawit Hailemariam Yesho, Muluken Zeleke Megiso, Chidiebube Ugwu, Ariana N. Neely, Tarfa Verinumbe, Gabor Varadi, Colton Jones

**Affiliations:** 1 Department of Internal Medicine, Jefferson Einstein Philadelphia Hospital, Philadelphia, PA USA; 2 Department of Hematology and Oncology, Jefferson Einstein Philadelphia Hospital, Philadelphia, PA USA; 3 The University of Texas, San Antonio, San Antonio, TX USA

**Keywords:** Breast cancer, Postmastectomy lymphedema, Coded lymphedema, GLP-1 receptor agonist, Survivorship, Obesity, Real-world data

## Abstract

**Purpose:**

Postmastectomy lymphedema is a chronic survivorship complication after breast cancer treatment, particularly among patients exposed to axillary surgery. Obesity and metabolic inflammation are recognized contributors to lymphedema risk. This study evaluated whether glucagon-like peptide − 1 receptor agonist exposure was associated with lower incidence of coded postmastectomy lymphedema among breast cancer patients with mastectomy and/or axillary procedure exposure.

**Methods:**

We performed a retrospective cohort study using deidentified electronic health record data from the TriNetX Research Network. Adult patients with breast cancer and coded mastectomy and/or axillary procedural exposure were stratified by GLP-1 receptor agonist exposure versus no exposure. One-to-one propensity score matching was used to balance measured baseline characteristics. The primary outcome was incident coded postmastectomy lymphedema syndrome. Outcomes were assessed from 1 to 650 days after the index event using risk estimates, Kaplan–Meier analysis, and Cox proportional hazards models.

**Results:**

After propensity score matching, 61,630 patients were included, with 30,815 patients in each cohort. Coded postmastectomy lymphedema occurred in 258 of 29,259 GLP-1 receptor agonist users and 779 of 30,237 non-users, corresponding to incidence rates of 0.88% and 2.58%, respectively. GLP-1 receptor agonist exposure was associated with lower incidence of coded postmastectomy lymphedema in time-to-event analysis (hazard ratio 0.373, 95% CI 0.324–0.429; p < 0.001). The absolute risk difference was − 1.7%, with an estimated number needed to treat of 59.

**Conclusion:**

In this large real-world breast cancer surgical/procedural cohort, GLP-1 receptor agonist exposure was associated with lower incidence of coded postmastectomy lymphedema over 650 days of follow-up. These findings are hypothesis-generating and require prospective validation with systematic lymphedema assessment and detailed characterization of GLP-1 receptor agonist timing, duration, dose, and adherence.

## Introduction

Breast cancer-related lymphedema remains one of the most persistent complications of breast cancer survivorship. It can cause arm swelling, heaviness, pain, reduced range of motion, recurrent infection, and impaired quality of life [1, 2]. As breast cancer outcomes improve and survivorship populations grow, prevention of chronic treatment-related morbidity has become an increasingly important goal in supportive oncology.

The reported incidence of breast cancer-related lymphedema varies across studies because of differences in diagnostic criteria, measurement techniques, treatment exposures, and duration of follow-up [1, 2]. In a systematic review and meta-analysis, DiSipio and colleagues reported that more than one in five breast cancer survivors developed arm lymphedema, with risk increasing over the first two years after treatment [1]. Axillary lymph node dissection remains one of the strongest treatment-related risk factors, and the combination of axillary surgery with regional nodal irradiation further increases risk [3, 4]. Although sentinel lymph node biopsy has reduced the burden of lymphedema compared with more extensive axillary surgery, it has not eliminated the problem, particularly among patients receiving multimodality therapy [2–4].

Patient-level factors are also central to lymphedema risk. Elevated body mass index, obesity, and postoperative weight gain have been repeatedly associated with higher risk of breast cancer-related lymphedema and worse outcomes after lymphedema develops [1, 2, 5]. Obesity may impair lymphatic function through increased lymphatic load, adipose tissue inflammation, impaired lymphatic pumping, lymphatic endothelial dysfunction, and progressive fibroadipose remodeling [5–7]. These mechanisms are relevant because lymphedema is not simply passive fluid accumulation; it is increasingly understood as a chronic inflammatory and fibroadipose disorder of the lymphatic microenvironment [6, 7].

Current prevention strategies focus on surgical de-escalation when oncologically appropriate, prospective surveillance, early compression intervention, exercise, and weight optimization [2, 8–14]. Prospective surveillance programs using bioimpedance spectroscopy or limb-volume monitoring can identify early fluid changes before progression to chronic lymphedema [8, 9]. Progressive resistance training appears safe among patients with or at risk for breast cancer-related lymphedema [10, 11]. Weight-loss interventions have also been studied, although results have varied based on timing, intervention design, adherence, and whether lymphedema is already established [12–14]. Despite these advances, no pharmacologic therapy is currently established for primary prevention of postmastectomy lymphedema.

Glucagon-like peptide − 1 receptor agonists have become increasingly used for diabetes, obesity, and cardiometabolic risk reduction. Semaglutide and related agents can produce substantial weight loss and have demonstrated cardiovascular benefit in selected populations [15, 16]. Beyond weight reduction, GLP-1 receptor agonists may exert anti-inflammatory effects, including reduced macrophage infiltration and modulation of inflammatory signaling in adipose tissue [17, 18]. These properties provide a biologically plausible rationale for evaluating GLP-1 receptor agonists in lymphedema prevention, particularly in patients whose lymphatic function may already be compromised by surgery, radiation, obesity, or metabolic inflammation.

Direct evidence linking GLP-1 receptor agonists to cancer-related lymphedema is limited but emerging. A case report described improvement in breast cancer-related lymphedema after GLP-1 receptor agonist initiation [19]. A subsequent retrospective study suggested that GLP-1 receptor agonist use may reduce the risk of cancer-related lymphedema following axillary lymph node dissection [20]. However, prior evidence has been limited by small sample sizes, single-center design, and limited evaluation of postmastectomy lymphedema as a primary prevention endpoint in a large breast cancer population.

We therefore conducted a multicenter real-world cohort study using a large federated electronic health record network to evaluate whether GLP-1 receptor agonist exposure was associated with lower incidence of coded postmastectomy lymphedema among breast cancer patients with mastectomy and/or axillary procedure exposure. We hypothesized that GLP-1 receptor agonist exposure would be associated with lower coded postmastectomy lymphedema incidence during follow-up.

## Methods

### Study design and data source

We performed a retrospective cohort study using the TriNetX Research Network, a federated health research network containing deidentified electronic health record data from participating healthcare organizations. At the time of analysis, the network included 113 healthcare organizations. Available data included demographic characteristics, diagnoses, procedures, medications, and laboratory values. The analysis was conducted using the TriNetX Compare Outcomes workflow.

This study used aggregated, deidentified data. Because no direct patient contact occurred and no identifiable private health information was accessed, the study was considered exempt from institutional review board review/not human subjects research. Final institutional determination language and reference number, if applicable, should be inserted before submission.

### Study population

Eligible patients were adults with breast cancer, identified by diagnosis code for malignant neoplasm of the breast and/or oncology-domain breast cancer coding. Patients were required to have evidence of breast cancer surgery or axillary procedural exposure after breast cancer diagnosis. Qualifying criteria included mastectomy procedures, axillary lymphadenectomy, sentinel lymph node biopsy, or acquired absence of breast and nipple. Therefore, the cohort should be interpreted as a breast cancer surgical/procedural cohort with mastectomy and/or axillary procedure exposure rather than a cohort limited exclusively to patients undergoing mastectomy alone.

Patients were divided into two cohorts. The exposed cohort included breast cancer patients meeting the above surgical/procedural criteria with documented GLP-1 receptor agonist exposure. The comparator cohort included patients meeting the same breast cancer and surgical/procedural criteria without documented GLP-1 receptor agonist exposure.

### Exposure definition

The exposure of interest was documented receipt of a GLP-1 receptor agonist or related incretin-based agent in the electronic health record. Medications included semaglutide, liraglutide, dulaglutide, exenatide, lixisenatide, albiglutide, tirzepatide, and the GLP-1 analogue medication class. Exposure was treated as a binary variable in the primary analysis. The available aggregate data did not allow reliable characterization of medication dose, duration, adherence, persistence, indication, or cumulative exposure. The exact timing of GLP-1 receptor agonist initiation relative to breast cancer surgery and lymphedema diagnosis could not be adjudicated at the individual-patient level.

### Index date and follow-up

The index date was defined within the TriNetX Compare Outcomes workflow as the date on which a patient first met the selected cohort-defining criteria. Outcomes were assessed beginning 1 day after the index event and continuing through 650 days of follow-up. Patients with the outcome recorded before the start of the outcome window were excluded from the corresponding outcome analysis to reduce inclusion of prevalent lymphedema.

### Outcome definition

The primary outcome was incident coded postmastectomy lymphedema syndrome, defined by ICD-10-CM code I97.2. A secondary lymphedema outcome was defined as lymphedema not elsewhere classified, using ICD-10-CM code I89.0. Because outcomes were based on diagnosis codes rather than prospective surveillance, limb-volume measurements, bioimpedance spectroscopy, or clinical adjudication, the outcome should be interpreted as coded lymphedema rather than systematically confirmed clinical lymphedema.

### Propensity score matching

One-to-one propensity score matching was performed within the TriNetX platform to reduce measured confounding between GLP-1 receptor agonist users and non-users. Matching variables included demographic characteristics, diagnostic categories, medication classes, and available laboratory/metabolic variables. Standardized mean differences were used to assess covariate balance after matching.

Because GLP-1 receptor agonist users differed clinically from non-users before matching, particular attention was given to residual imbalance in metabolic variables after matching, including body mass index, body weight, and hemoglobin A1c. These variables were considered important potential sources of residual confounding and were addressed in the interpretation of the findings.

### Statistical analysis

Baseline characteristics were summarized using means with standard deviations for continuous variables and counts with percentages for categorical variables. Risk estimates were calculated as the proportion of patients developing the outcome during follow-up. Risk differences, risk ratios, odds ratios, and number needed to treat were calculated for the primary outcome.

Time-to-event analyses were performed using Kaplan–Meier methods. Cox proportional hazards models were used to estimate hazard ratios with 95% confidence intervals. Statistical significance was assessed using two-sided p values, with *p* < 0.05 considered statistically significant. All analyses were conducted within the TriNetX platform.

## Results

### Cohort characteristics

Before propensity score matching, the propensity-score eligible cohorts included 30,970 patients in the GLP-1 receptor agonist cohort and 376,922 patients in the non-exposed comparator cohort. After one-to-one propensity score matching, 61,630 patients were included, with 30,815 patients in each cohort.

The matched GLP-1 receptor agonist cohort had a mean age of 62.1 ± 10.8 years and was 99.2% female. The matched comparator cohort had a mean age of 62.5 ± 11.8 years and was also 99.2% female. Racial distribution was similar after matching, including 71.0% White patients and 18.0% Black or African American patients in the GLP-1 receptor agonist cohort compared with 72.2% White patients and 18.0% Black or African American patients in the comparator cohort.

Propensity score matching resulted in close balance across demographic variables, with standardized mean differences below 0.1 for age, sex, and race. However, residual imbalance persisted in measured metabolic variables. Mean body mass index remained higher in the GLP-1 receptor agonist cohort than in the comparator cohort, as did body weight and hemoglobin A1c. Baseline characteristics after propensity score matching are summarized in Table 1.

**Table 1:** Baseline characteristics after propensity score matching. Values are presented as mean ± standard deviation or *n* (%). Percentages are calculated within each matched cohort

Characteristic	Mastectomy + GLP-1 RA, *n* = 30,815	Mastectomy not on GLP-1 RA, *n* = 30,815	*p* value	Standardized mean difference
Age at index, years	62.1 ± 10.8	62.5 ± 11.8	< 0.001	0.037
Sex				
Female	30,577 (99.2%)	30,583 (99.2%)	0.781	0.002
Male	226 (0.7%)	220 (0.7%)	0.776	0.002
Race				
White	21,873 (71.0%)	22,253 (72.2%)	0.001	0.027
Black or African American	5,559 (18.0%)	5,559 (18.0%)	1.000	< 0.001
Asian	904 (2.9%)	753 (2.4%)	< 0.001	0.030
Other race	975 (3.2%)	882 (2.9%)	0.028	0.018
Unknown race	1,151 (3.7%)	1,091 (3.5%)	0.197	0.010
Available metabolic variables				
Hemoglobin A1c, %	7.3 ± 1.8	6.3 ± 1.4	< 0.001	0.597
Patients with available HbA1c	15,344 (49.8%)	15,154 (49.2%)	0.126	0.012
Body mass index, kg/m2	35.3 ± 7.2	30.1 ± 7.3	< 0.001	0.720
Patients with available BMI	22,343 (72.5%)	22,240 (72.2%)	0.326	0.008
Body weight, lb	206.5 ± 45.6	175.5 ± 45.3	< 0.001	0.682
Patients with available body weight	21,380 (69.4%)	21,344 (69.3%)	0.753	0.003

*GLP-1 RA* Glucagon-like peptide − 1 receptor agonist, *HbA1c* Hemoglobin A1c

Propensity score matching resulted in close balance across demographic variables. Residual imbalance remained in measured metabolic variables, including HbA1c, BMI, and body weight

### Primary outcome: Postmastectomy lymphedema

After excluding patients with coded postmastectomy lymphedema recorded before the outcome window, 29,259 GLP-1 receptor agonist users and 30,237 non-users were included in the primary outcome analysis. Coded postmastectomy lymphedema occurred in 258 patients in the GLP-1 receptor agonist cohort and 779 patients in the comparator cohort. The corresponding coded incidence rates were 0.88% and 2.58%, respectively. The incidence of coded postmastectomy lymphedema by cohort is summarized in Table 2.

**Table 2:** Incidence of coded postmastectomy lymphedema after propensity score matching

Cohort	Patients with outcome, n/N	Patients with outcome, %	*p* value
Mastectomy + GLP-1 RA	258/29,259	0.88%	< 0.0001
Mastectomy not on GLP-1 RA	779/30,237	2.58%	< 0.0001

Outcome: coded postmastectomy lymphedema

*GLP-1 RA* Glucagon-like peptide − 1 receptor agonist

GLP-1 receptor agonist exposure was associated with a lower incidence of coded postmastectomy lymphedema. The absolute risk difference was − 1.7% (95% CI − 1.9% to − 1.5%), corresponding to an estimated number needed to treat of 59. The risk ratio was 0.342, and the odds ratio was 0.336. In Kaplan–Meier analysis, lymphedema-free survival at 650 days was 98.87% in the GLP-1 receptor agonist cohort and 97.16% in the comparator cohort. Cox proportional hazards analysis showed a hazard ratio of 0.373 (95% CI 0.324–0.429; *p* < 0.001).

### Secondary lymphedema outcome

For the broader lymphedema outcome, excluding patients with prior lymphedema before the outcome window, lymphedema occurred in 1,021 of 25,926 GLP-1 receptor agonist users and 2,454 of 29,092 non-users. The corresponding coded incidence rates were 3.9% and 8.4%, respectively. GLP-1 receptor agonist exposure was associated with lower incidence of the broader coded lymphedema outcome, with a hazard ratio of 0.507 (95% CI 0.472–0.546; p < 0.001).

## Discussion

In this large multicenter real-world cohort of breast cancer patients with mastectomy and/or axillary procedure exposure, GLP-1 receptor agonist exposure was associated with lower incidence of coded postmastectomy lymphedema over 650 days of follow-up. The association was observed in both risk-based and time-to-event analyses. The absolute difference in coded postmastectomy lymphedema incidence was 1.7%, corresponding to an estimated number needed to treat of 59. A similar direction of association was observed for the broader coded lymphedema outcome.

These findings extend a growing body of literature linking metabolic health, obesity, inflammation, and lymphatic dysfunction. Obesity is a recognized risk factor for breast cancer-related lymphedema, and several mechanisms may contribute to this association, including increased lymphatic load, adipose tissue inflammation, lymphatic endothelial dysfunction, and progressive fibroadipose remodeling [5–7]. GLP-1 receptor agonists may plausibly influence these pathways through weight reduction, improved glycemic control, and anti-inflammatory effects [15–18]. However, the present study was not designed to determine the mechanism of association, and actual weight change after GLP-1 receptor agonist initiation was not evaluated in the primary analysis.

Our findings also align with early clinical observations suggesting a potential role for GLP-1 receptor agonists in cancer-related lymphedema. Crowley and colleagues reported improvement in breast cancer-related lymphedema after GLP-1 receptor agonist therapy in a case report [19]. Brown and colleagues subsequently reported that GLP-1 receptor agonist use was associated with reduced cancer-related lymphedema risk after axillary lymph node dissection [20]. The present study expands on this early evidence by evaluating a substantially larger multicenter cohort, while also differing in that it examined a broader breast cancer surgical/procedural population rather than an ALND-restricted cohort.

The findings should be interpreted within the context of contemporary breast cancer survivorship care. Current lymphedema prevention strategies rely primarily on surgical de-escalation when oncologically appropriate, prospective surveillance, early compression intervention, exercise, and weight optimization [2, 8–14]. These approaches are important but do not fully address the biologic interaction between metabolic inflammation and lymphatic injury. If the observed association between GLP-1 receptor agonist exposure and coded postmastectomy lymphedema is confirmed prospectively, metabolic interventions could become an area of further study for lymphedema risk reduction in selected high-risk patients.

This study has several strengths. It used a large multicenter federated electronic health record network, included more than 60,000 matched patients, excluded patients with prior outcome codes before the outcome window, and evaluated both risk-based and time-to-event estimates. The study also focused on a clinically important survivorship endpoint rather than tumor response or cancer control, making the question directly relevant to supportive oncology.

Several limitations should be emphasized. First, this was an observational study and cannot establish causality. Second, the cohort was defined using breast cancer diagnosis plus mastectomy and/or axillary procedural codes, including mastectomy procedures, axillary lymphadenectomy, sentinel lymph node biopsy, and acquired absence of breast and nipple. As a result, the cohort was not limited exclusively to patients undergoing mastectomy with nodal surgery. Patients with mastectomy alone or less extensive axillary intervention may have a lower baseline risk of breast cancer-related lymphedema than patients undergoing axillary lymph node dissection or combined-modality treatment. Their inclusion may have diluted the overall observed coded lymphedema incidence and limits direct comparison with studies restricted to axillary lymph node dissection populations.

Third, the primary outcome was based on coded diagnosis of postmastectomy lymphedema rather than systematic prospective lymphedema surveillance, objective arm measurements, bioimpedance spectroscopy, or clinical adjudication. The incidence observed in this study was substantially lower than rates reported in prospective surveillance studies, including reports of approximately 8–10% lymphedema incidence even after sentinel lymph node biopsy [3]. This suggests that coded electronic health record diagnoses likely underestimate the true incidence of breast cancer-related lymphedema. If undercoding differed between GLP-1 receptor agonist users and non-users, the observed association could be biased. Even if undercoding was nondifferential, reliance on diagnosis codes likely reduced outcome sensitivity and limits interpretation of the absolute event rates.

Fourth, GLP-1 receptor agonist exposure was treated as a binary variable. The analysis did not capture medication dose, duration, adherence, persistence, discontinuation, indication for treatment, or cumulative exposure. A patient with a brief or unfilled prescription could be classified similarly to a patient receiving long-term therapy. In addition, the aggregate workflow did not allow detailed individual-level adjudication of GLP-1 receptor agonist timing relative to breast cancer surgery and subsequent lymphedema diagnosis. These limitations are important when interpreting a temporal association between GLP-1 receptor agonist exposure and coded lymphedema.

Fifth, although propensity score matching improved balance across many measured characteristics, residual imbalance remained in key metabolic variables, including body mass index, body weight, and hemoglobin A1c. These differences may reflect confounding by indication, because GLP-1 receptor agonist users were more likely to have obesity, diabetes, or metabolic disease. Sixth, the analysis could not fully account for breast cancer stage, radiation fields, number of lymph nodes removed, reconstruction type, endocrine therapy duration, chemotherapy regimen, taxane exposure, or longitudinal weight change. Seventh, the 650-day follow-up period may not capture all incident lymphedema events, because breast cancer-related lymphedema can develop years after treatment. Finally, procedure-specific, medication-specific, obesity-stratified, and diabetes-stratified analyses were not performed in the present study and should be prioritized in future work.

Despite these limitations, the magnitude and consistency of the association support further study. Prospective observational studies with detailed oncologic treatment variables, objective lymphedema measurements, and longitudinal weight data would help clarify the relationship. Ultimately, randomized or pragmatic intervention studies may be needed to determine whether GLP-1 receptor agonists directly reduce clinically confirmed postmastectomy lymphedema risk and which patients are most likely to benefit.

## Conclusion

In a large multicenter real-world cohort of breast cancer patients with mastectomy and/or axillary procedure exposure, GLP-1 receptor agonist exposure was associated with lower incidence of coded postmastectomy lymphedema over 650 days of follow-up. These findings are hypothesis-generating and should not be interpreted as evidence that GLP-1 receptor agonists prevent clinically confirmed lymphedema. Prospective studies are needed with rigorous characterization of GLP-1 receptor agonist dose, duration, timing, adherence, and indication, together with systematic and objective lymphedema assessment.

## Data Availability

No datasets were generated or analysed during the current study.

## Declarations

### Use of AI-assisted tools

AI-assisted tools were used to support language organization and editorial refinement. All authors reviewed, revised, and verified the manuscript content, data interpretation, and references, and take full responsibility for the final submitted work. No AI tool was used to perform the statistical analysis. [Include, revise, or remove based on journal policy, institutional guidance, and coauthor preference.]
